# Discovery of microRNA-target modules of African rice (*Oryza glaberrima*) under salinity stress

**DOI:** 10.1038/s41598-017-18206-z

**Published:** 2018-01-12

**Authors:** Tapan Kumar Mondal, Alok Kumar Panda, Hukam C. Rawal, Tilak Raj Sharma

**Affiliations:** 1 Division of Genomic Resources, ICAR-National Bureau of Plant Genetic Resources, Pusa, IARI Campus, New Delhi, 110012 India; 2 ICAR-National Research Centre on Plant Biotechnology, L.B.S. Building, IARI Campus, New Delhi, 110012 India

## Abstract

*Oryza glaberrima* is the second edible rice in the genus Oryza. It is grown in the African countries. miRNAs are regulatory molecules that are involved in every domains of gene expression including salinity stress response. Although several miRNAs have been reported from various species of *Oryza*, yet none of them are from this species. Salt treated (200 mM NaCl for 48 h) and control smallRNA libraries of RAM-100, a salt tolerant genotype, each with 2 replications generated 150 conserve and 348 novel miRNAs. We also used smallRNAseq data of NCBI of *O*. *glaberrima* to discover additional 246 known miRNAs. Totally, 29 known and 32 novel miRNAs were differentially regulated under salinity stress. Gene ontology and KEGG analysis indicated several targets were involved in vital biological pathways of salinity stress tolerance. Expression of selected miRNAs as indicated by Illumina data were found to be coherent with real time-PCR analysis. However, target gene expression was inversely correlated with their corresponding miRNAs. Finally based upon present results as well as existing knowledge of literature, we proposed the miRNA-target modules that were induced by salinity stress. Therefore, the present findings provide valuable information about miRNA-target networks in salinity adaption of *O*. *glaberrima*.

## Introduction

African rice (*Oryza glaberrima* Steud), is the second edible rice next to *O*. *sativa* L., a native of sub-Saharan Africa. This has been domesticated around 2000–3000 years ago from its wild ancestor *O*. *barthii* by the inhabitants of the floodplains, near the bend of the Niger river^1^. In general, it has pear-shaped red-bran grains with an olive-to-black seed coat, short-branched panicles with either rounded or pointed ligules^2^. It also offers distinct advantages due to its inherent tolerance to various stresses including salinity. Specifically, African rice is tolerant to flooding, infertile soil, iron toxicity and human neglects. Few of the genotypes mature earlier than Asian rice and hence suitable for short duration crops^3,4^.

High salinity stress is an important abiotic factor that reduces rice production^5,6^. Rice though a major cereal yet sensitive to salinity stress. Salt accumulation of irrigated soil is major cause that reduces soil water to root-cells of rice plants due to the reduction of osmotic potential which ultimately diminish the growth, development and yield of rice^7,8^. Therefore, it is essential to develop salinity stress tolerant rice genotypes to minimize salinity related yield penalty^9–11^. However, the pre-requisite for salinity stress tolerant breeding is to discover the genomic resources that are related to salinity stress tolerance. While salt responsive genomic resources from rice have been extensively studied, their orthologs from wild species of rice are largely unexplored. Plants express several genes including various non-coding RNAs such as miRNAs under unfavourable environmental conditions, which have well-defined molecular, biochemical and physiological functions^12^, to combat adverse situation.

Small endogenous non-coding RNAs are important for modulating the gene expression by regulating their targets, in diverse biological and moleculer processes such as organ development, signalling, tolerant to various biotic and abiotic stresses^13,14^. Several stress-responsive novel and known miRNAs including salinity stress of many plants, have been identified and characterized^15–17^.

High-throughput sequencing is highly efficient method that is used for miRNA discovery^18^. It has fasten the discovery of sRNAs from several plant species due to its cost-effectiveness, capability to discover novel miRNAs, high speed to sequence particularly for orphan species^18,19^. Thus, we have used high-throughput sequencing to discover miRNA-target modules from *O*. *glaberrima* from a salt tolerant genotype, under salinity stress.

## Results

### Analysis of sequencing reads

**Figure 1:**
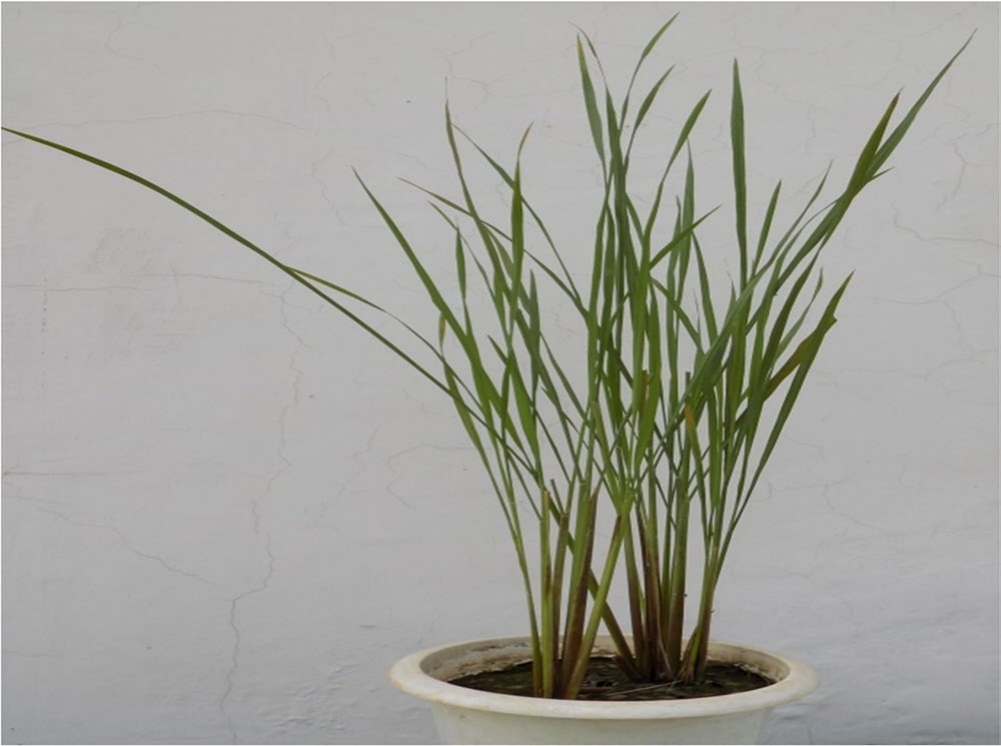
Two-month old seedlings of *O*. *glaberrima* growing in pot, young leaf of which was taken for preparation of small RNAseq library.

For the present study, we used a salt tolerant genotype of *O*. *glaberrima* (Fig. 1). To identify genome-wide and subsequently salinity stress tolerant responsive miRNAs of *O*. *glaberrima*, two smallRNA libraries each with two replications were sequenced from both non-treated as well as salt-treated sample. This generated 20451980 and 23806967 raw reads from the non-treated and treated samples, respectively. Subsequently selection of size, exclusion of adapters and other non-coding RNA sequences, 1598031 clean reads turned to be 1204677 unique reads in control library and 16570811 clean reads to 1084658 unique reads in salinity stressed. These results are tabulated in Table 1.

**Table 1:** Classification of different reads between two libraries*.

Name of the reads	Control	Treated
Raw reads	20451980	23806967
Clean reads	1598031	16570811
Unique reads	1204677	1084658
Reads mapped to miRbase	738381	765241.5
Unique reads	435782	409876
Known miRNAs	134	130
Reads without annotation	755721.5	729209
Unique reads	316752	287673
Novel miRNAs	271	179

*The values are average of the two replicates.

**Figure 2:**
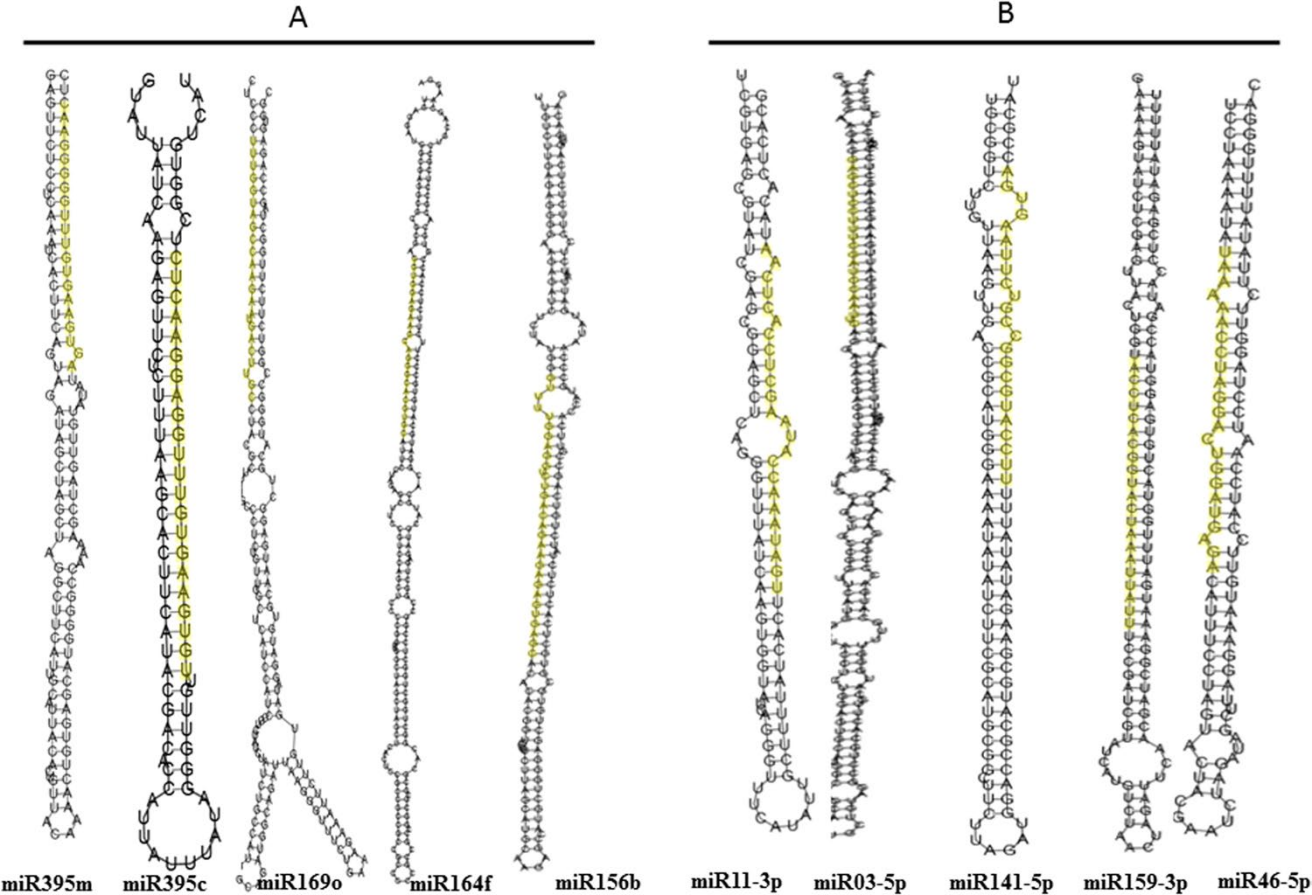
Stem-loop structure of few representative miRNAs of *O*. *glaberrima*. (**A**) miRNAs predicted through *in silico* analysis, (**B**) Novel predicted miRNAs (high lights indicate the position of miRNA).

The reads were subsequently mapped to the *O*. *glaberrima* genome by SOAP 2^20^. Finally, the reads of conserved non-coding RNAs such as snRNA, siRNA, tRNA, rRNA, snoRNA, intron as well as exon were discarded (Fig. S2). Subsequently, 738381 (unique reads 435782) in control and 765241 (unique reads 409876) in salinity stressed library were mapped to the miRBase respectively (Table 1). Further, 755721 reads in control and 729209 reads in stressed libraries were found to be unannotated due to their uniqueness to *O*. *glaberrima* genome. Among the clean reads, maximum number was 24 nt long (Fig. S3) and then 21 nt, which are reported in literature^21–23^, including wild species of rice^21,24^. This may be because selective cleavage of pre-miRNA sequences by DCL1 produce 21 nt and DCL3 as well as DCL4 produce 24 nt miRNAs. Variations of different length of mature miRNAs make them suitable for RISC complex to control target gene expression. Some of the predicted precursors structure are shown in Fig. 2. Importantly, in control library, 85 and 49 known miRNAs and in salt-treated library, 80 and 50 known miRNAs were originated from sense and anti-sense strand respectively.

### Discovery of known miRNAs

**Figure 3:**
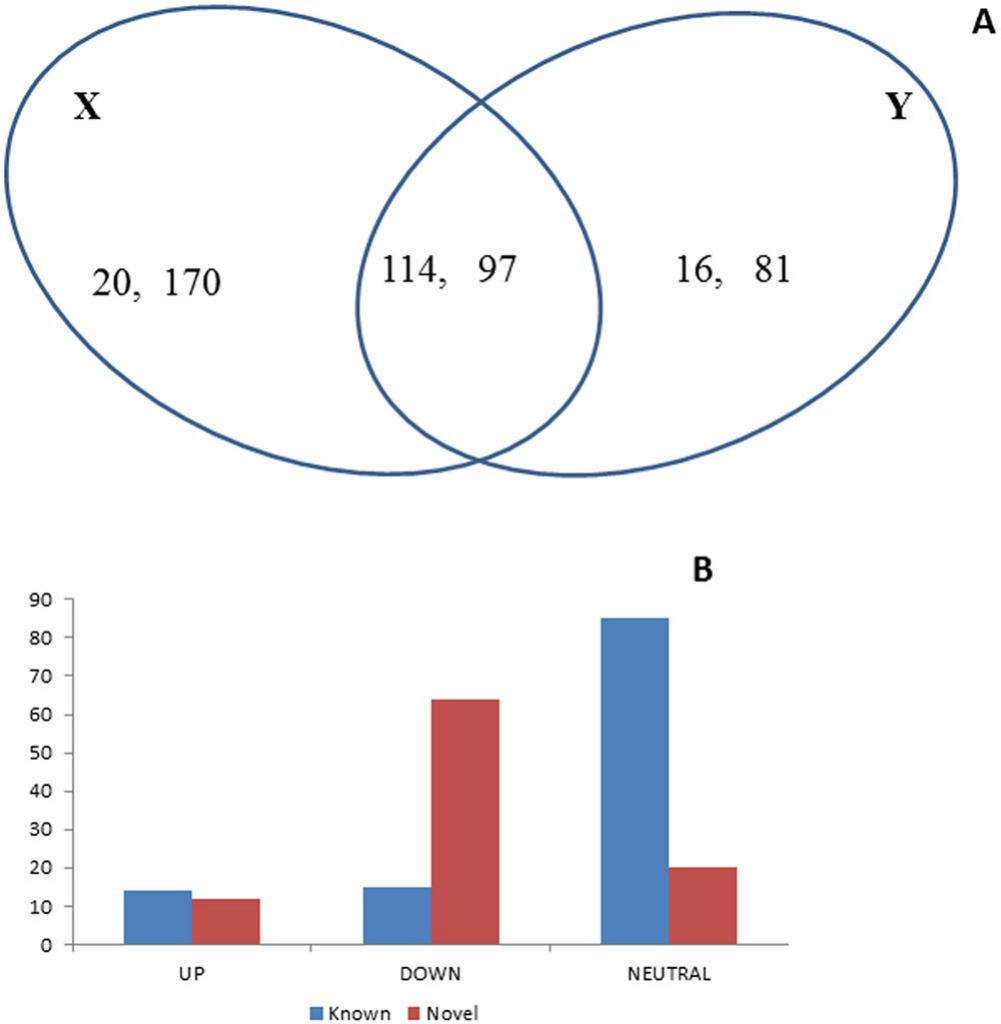
Number of miRNAs of *O*. *glaberrima*. (**A**) Total number of miRNAs of *O*. *glaberrima*. X = Control Y = 48 h salt-treated. The first and second numerical indicate known and novel miRNAs respectively. (**B**) Differential expressed miRNAs that are found in both the libraries. Y axis indicates the number of miRNAs.

Based upon sequence homology search by BLASTN with rice miRNAs of miRBase 21.0^25^, we identified, a total of 148 known miRNAs of *O*. *glaberrima*, of which 114 were found to be common (Fig. 3A; Table S2). These known miRNAs consisted of 66 and 63 different families in control library and salinity stressed library respectively (Table S3). Known miRNAs reads were found to be from 1 to 586088 in the non-treated library and from 1 to 275828 in stressed library indicating that there are varying level of expression of these miRNAs in both the libraries (Tables S2 and S3) which is also reported in the literature^26^. Besides, length of the mature miRNAs (20 nt to 25 nt) as well as precursor miRNAs (69 nt to 377 nt) was also varied. Some of the family and few members of some family, were found to be unique which are also reported in the literature^27^. Additionally, due to higher sensitivity of high throughput sequencing technology, it was possible to detect 3p-miRNAs sequences in both libraries respectively. However, most of the miRNAs had up to maximum 20 reads. This may be due to the degradation of pre-miRNA during the formation of mature miRNAs^28^. Some miRNAs such as ogl-miR156l, ogl-miR166c, ogl-miR166k, ogl-miR168a, ogl-miR167i, ogl-miR171f, ogl-miR1846d of control library and ogl-miR408, ogl-miR528, ogl-miR156, ogl-miR390, ogl-miR396c of treated library had higher reads than their complementary stand, respectively (Tables S2 and S3). For an example, in control library ogl-miR166k has only 2 reads whose complementary sequence i.e. ogl-miR166k-3p has 8654 reads. Similar results were also found in literature^24,29^. This is because they both i.e. miRNA and its complementary sequence i.e. miRNA* may function simultaneously for regulating gene expression.

### Discovery of novel miRNAs

**Figure 4:**
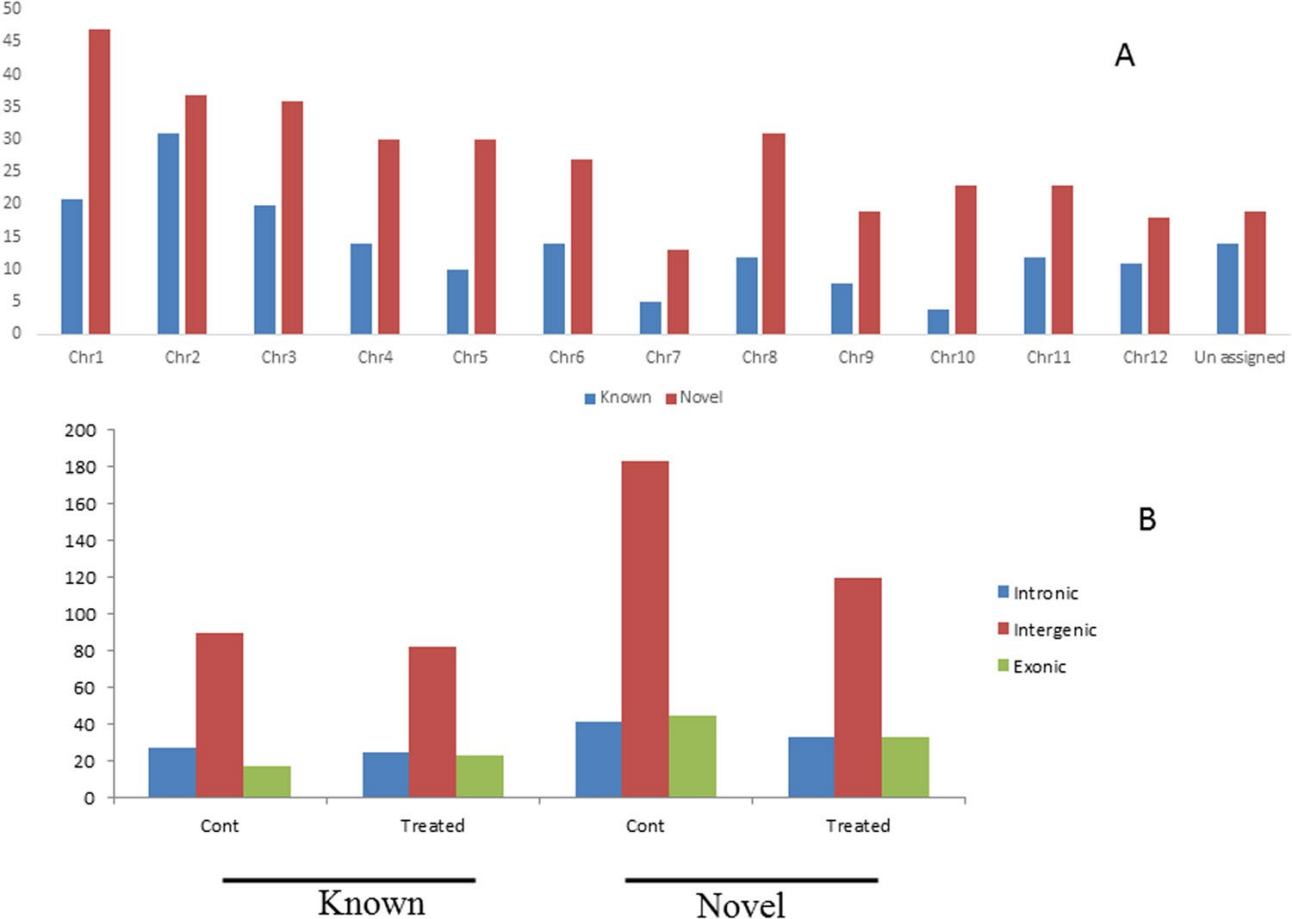
Distribution of novel miRNAs in *O*. *glaberrima* genome. (**A**) Chromosomal location of miRNAs. Y axis represents the number of miRNAs. (**B**) Within chromosome locations of different novel miRNAs. Y axis represents the number of miRNAs.

Further, to identify the novel miRNAs, unique sRNA sequences were mapped on its genome and subsequently stem-loop of the pre-cursor RNAs were determined (Fig. 2). In addition to that, binding sequences of dicer enzymes and the free energy of pre-miRNA sequences were also determined. This way, we have discovered total 348 novel miRNAs, out of which 97 miRNAs were found in both the libraries. Among them, 267 and 178 novel miRNAs were identified in the non-treated and salinity-stressed library, respectively (Fig. 3A; Table 1; Tables S4 and S5). Interestingly, these miRNAs had different reads reflecting their differential expression which may be either genotype or condition (salinity stress) specific (Tables S4 and S5). The precursors were found to be varied from 64 to 368 nt for non-treated library (Table S4) and 64 to 363 nt, for treated library (Table S5). Genomic location and orientation of the pre-miRNAs also varied. In the non-treated library, 137 as well as 134 novel miRNAs, and in salt-treated library 88 as well as 91 novel miRNAs were housed on sense and anti-sense orientation, respectively. Although, majority of them were single locus origin, yet same genetic locus produced more than one miRNA. They were 10 and 14 for non-treated and stressed library. Similar results were also found with that of *O*. *coarctata*^24^ and in *O*. *rufipogon*^29^. In total, there were 170 and 81 number of novel miRNAs found in non-treated and stressed library, respectively of which 97 were found to be common (Fig. 3A). We also found differences in location of miRNA loci (Fig. 4A). Interestingly, maximum number of miRNAs were housed in 1^st^ chromosome in both the libraries, whereas minimum number was found to be located in 12^th^ chromosome. Origin of the miRNAs within a gene was also differed. For an example, most of them were intergenic (183 and 120 miRNAs in non-treated and stressed library, respectively) followed by, 41 and 33 miRNAs in non-treated and salinity stressed library were intronic, respectively. Interestingly, 26 and 45 miRNAs were exonic in salt-treated as well as in control library, respectively (Fig. 4B; Tables S4 and S5). Occurrences of exonic miRNAs are also reported in rice, its wild species and Arabidopsis^30^. Although function of exonic miRNAs are poorly understand in plants, yet Hinske *et al*.^31^, suggested that they may potentially be involved in multiple layers of gene regulation in plants.

### Discovery of known miRNAs from published database

*In silico* identification is an alternative technique that detects known miRNAs sequences across the different taxa based upon sequence homology. From 6 RNAseq libraries of NCBI, we discovered 246 additional mature miRNAs along with their pre-cursor sequences that were absent in our present study (Fig. 2; Table S6). It has been found that those precursors had an average length of 146 nt, averaged free energy of −65.053 kcal/mol. Many of them are conserved across the plant species. This approach is widely used to identify and characterize known miRNAs of various plant species^32^. However their absence in our NGS data may be due to the fact that they are either genotype specific or could be developmentally regulated, a phenomenon which has been widely reported^33^. Comparative genomics through bioinformatics has been reported to identify known miRNAs in various plant species^32^, including rice^34^.

### Differentially expressed miRNAs

To analyse the expression, we studied the log_2_-ratios of known miRNA between the libraries (Table S7). Expression values in terms of fold change with ≥ or ≤ 1.0-fold due to salinity stress were taken. This analysis identified 85 neutral, 14 up-regulated (log2 ≥ +1, *p* 0.05) and 15 down-regulated (log2 ≤ −1, *p* 0.05) miRNAs. For an example, expression of some known miRNAs such as ogl-miR396a, ogl-miR529a, ogl-miR159b and ogl-miR397b, ogl-miR528-5p, ogl-miR167b had remarkable differences (Table S7). Similarly, 64 neutral, 12 up- and 20 down-regulated novel miRNAs were discovered (Fig. 3B; Table S8).

### Identification and expression of targets

Based upon the near perfect or perfect sequence complementary with mature miRNAs along with their targets, *in silico* target identification became easy^35^. In the present study, psRNATarget, Gene Ontology and KEGG were used to identify and characterize the targets of differentially expressed miRNAs. Accordingly, 29 conserved miRNAs of non-treated library produced 325 targets whereas 32 novel miRNAs had 350 targets (Tables S9 and S10). This is because majority of the miRNAs have multiple targets. On the other side, a single target also had multiple miRNAs. This is perhaps single target genesis involved in more than one stress. Average target genes varied from a minimum of 2 (ogl-miR137-5p; osa-miR398b) to maximum of 43 (ogl-miR07-5p) per miRNA. Importantly, target genes were functionally diversed e.g. enzymes, transcription factors and functional as well as regulatory proteins (Tables S9 and S10). On the basis of KEGG pathways analysis and existing knowledge of literature, 25 targets were found to be related to salinity stress tolerance. Hence, these 25 miRNA-target modules may be involve in salinity adaptation of this species.

**Figure 5:**
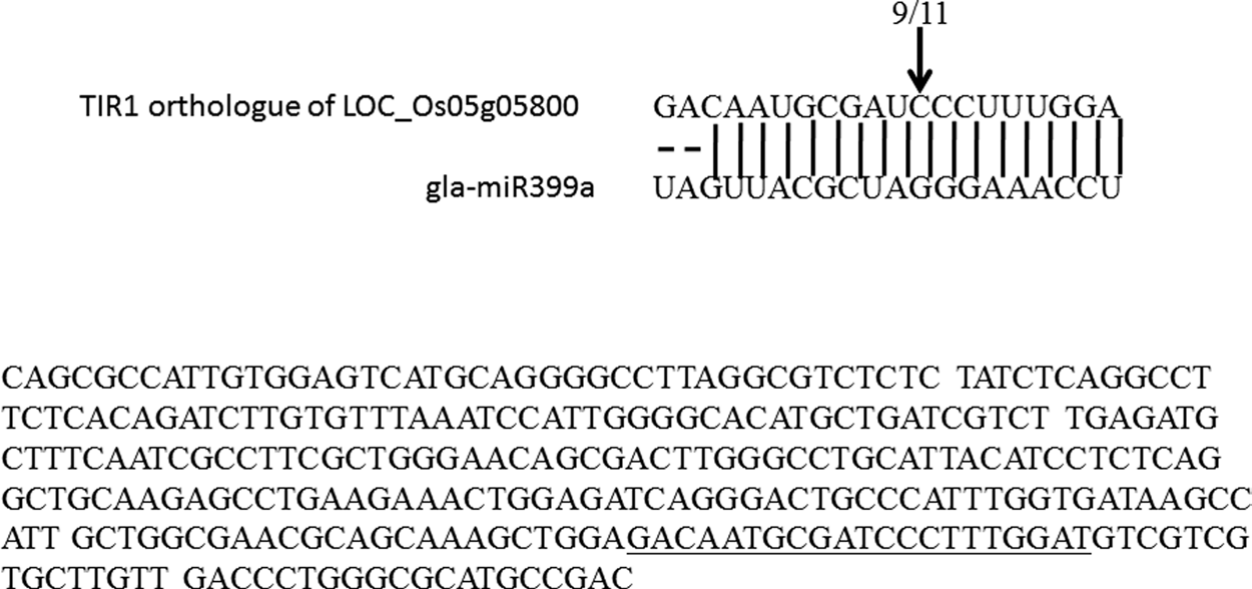
Target cleavage sites of miR393a by 5′ RLM-RACE. TIR1, the target of miR393a is an auxin receptor protein. The arrow depicts cleavage site, and the numbers above the arrow indicate the frequencies of the sequenced clones.

**Figure 6:**
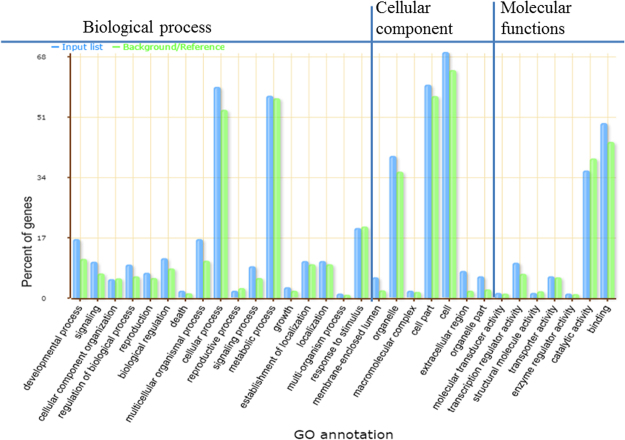
GO categories of target genes of known and new miRNA families. Targets were categorised according to the three domains of GO; biological process, cellular component and molecular function.

To demonstrate that ogl-miR393a may target TIR1, we used 5′RLM-RACE and confirmed that TIR1 was the target of ogl-miR393a. Sequences of 5′cleaved product of TIR1′ generated an accurate slice between the 10^th^ and 11^th^ base of ogl-miR393a from the 5′ end of target (Fig. 5). Earlier, this module was experimentally validated in rice^36^ and wild relative of rice^24^ under salinity stress indicating that this module is conserved at least across the *Oryza* species. Therefore, our results are similar with the published reports. Biological significance of the target genes of *O*. *glaberrima*, was further investigated by GO analysis^37^. Although genome sequence of *O*. *glaberrima* is available yet it is not well-annotated and hence we decided to do with rice orthologous genes to determine the GO term. Next, the targets were analysed with SEA in AgriGO to identify enriched GOs^37^. Genes of the enriched GO terms were analyzed by BLASTN against rice genome *O*. *sativa* MSU 7.0 which generated 117 significant GO terms (Fig. 6). For biological processes, 15 categories of genes were formed and 46 miRNA targets were found to be similar to metabolic processes (GO:0008152), followed by 24 targets were found to be similar to response to stimuli (GO:0050896) indicating that they may involve in various biological processes related to salinity stress tolerance mechanism. In Cellular Component Categories, targets were found in 5 different cellular parts, among them 3 most dominant terms were organelle (38), cell (46) and cell part (47). On the contrary, 3 most frequent GO terms in Molecular Function were found to be transcription regulator activity (19), catalytic activity (38) and binding (42) (Fig. 6). In addition, a large number of enriched GO terms in response to salt treatment was identified (Table S11 and Fig. S4). Further, for the reduction of GO terms, we used REVIGO (REduce and Visualize GO) program^38^. Taking Uniprot as reference dataset, we found that biological processes related with regulation of transcription and transportation, cellular homeostasis and cell death were dominant in this species that were related to salinity. Further, targets were analysed by KEGG pathway which generated 10 and 19 pathways in non-treated and salinity stressed libraries, respectively. Several pathways were found to be induced upon salinity stress. They were phenyl propanoid metabolism, tyrosine and tryptophan biosynthesis, biosynthesis of secondary metabolites, biosynthesis of ubiquitine and other terpenoid pathways, starch as well as sucrose metabolism, few of which were consistent with biological processes as determined by GO. In total, 25 targets were involved in KEGG pathway analysis (Table S12). Using KEGG functional annotations, target genes, regulated by miRNAs, were classified to identify the salinity stressed related pathways (Table S12) in the leaf tissue of *O*. *glaberrima*.

### qPCR analysis of miRNAs and their targets

**Figure 7:**
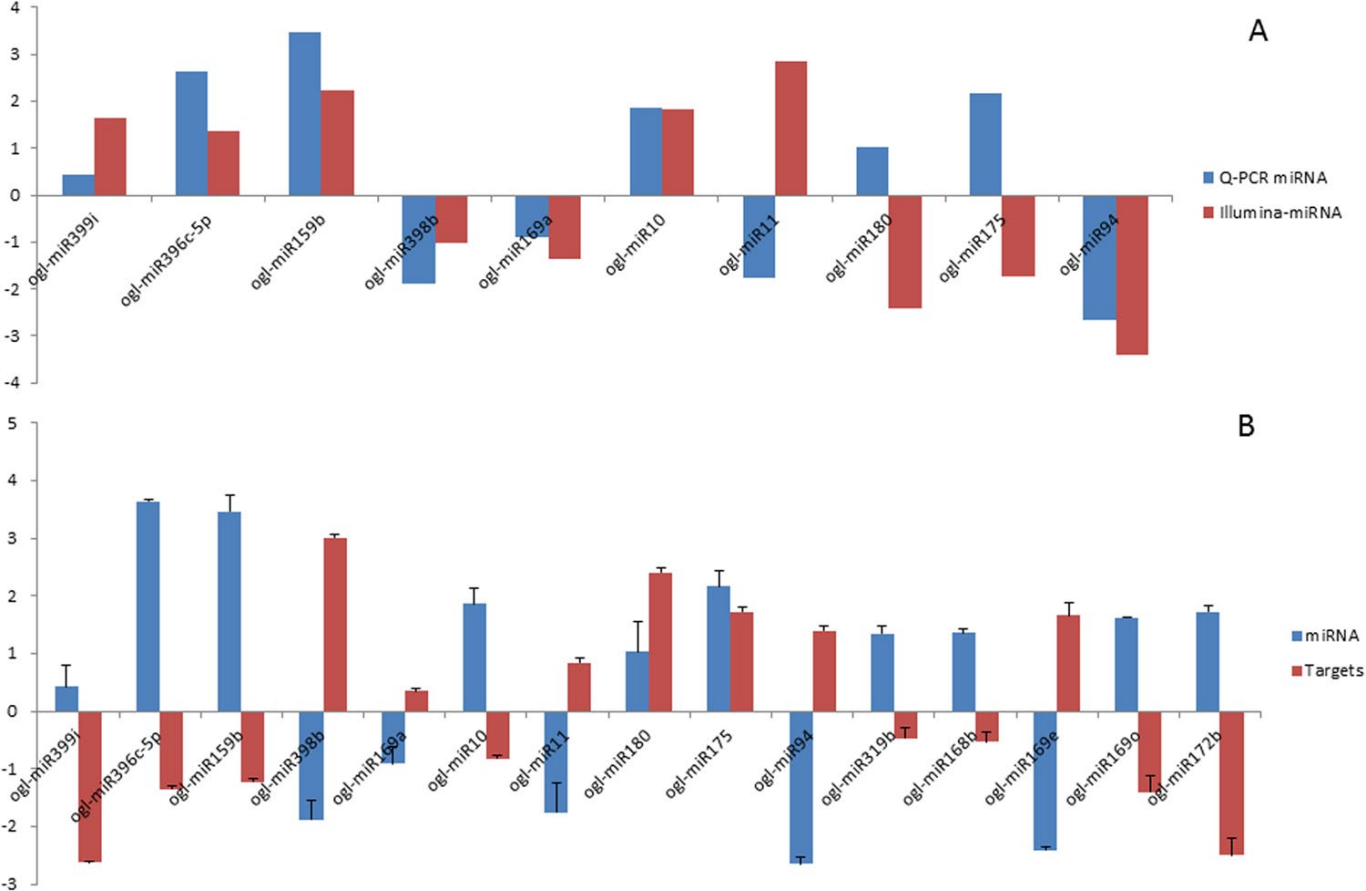
q-PCR analysis of miRNAs and targets: (**A**) Relative expression of miRNAs (fold changes of illumina reads and qRT-PCR) between control and salt-treated library. (**B**) Relative expression of miRNAs and their targets between control and salt-treated condition. The data represents the mean values ± SD of three replicates. Y axis represents the relative fold change.

For validating the results of our NGS data, we did qPCR analysis of 10 randomly selected differentially expressed miRNAs with their respective targets. It was seen that 7 out of the 10 miRNAs had the same pattern of expression as seen by both the analyses (Fig. 7A). These included ogl-miR399i, ogl-miR396c-5p, ogl-miR159b, ogl-miR10 which were up-regulated whereas, ogl-miR398b, ogl-miR169a and ogl-miR94 were down regulated under salinity stress by both the analysis i.e. qRT-PCR results as well as high throughput sequencing reads. On the other hand, ogl-miR11, ogl-miR180 and ogl-miR175 showed reverse expression patterns. Among the target genes, copper/zinc superoxide dismutase, putative, expressed (LOC_Os07g46990), nuclear transcription factor Y subunit, (LOC_Os12g42400) and calcium-binding mitochondrial protein(LOC_Os04g41950) for ogl-miR398b, ogl-miR169a and ogl-miR94, respectively, were up-regulated under salinity stress, while ataxin-2 C-terminal region family protein, (LOC_Os04g55230), jasmonate O-methyl transferase, (LOC_Os04g57050), MYB family transcription factor, putative (LOC_Os01g12700), calcium binding proteins (LOC_Os04g41950) for ogl-miR399i, ogl-miR396c-5p, ogl-miR159b, ogl-miR10 respectively were down-regulated under salinity stressed. Thus, these miRNAs i.e. ogl-miR398b, ogl-miR169a, ogl-miR94, ogl-miR399i, ogl-miR396c-5p, ogl-miR159b and ogl-miR10 are salt-responsive miRNAs (Fig. 7A). Zhao *et al*.^39^ also found that rice miR169 was up-regulated under salinity stress. Besides, miR396c^40^, miR398^41^, miR159^42^, are also found to be salt responsive miRNAs of rice.

Further, expression analysis of 15 miRNA-target modules showed reverse pattern for 13 indicating that they are the true targets for these miRNAs. However rest 2 miRNAs showed similar expression patters as indicated by qPCR results (Fig. 7B).

### Availability of novel miRNAs in selected monocots and dicots

We named 335 novel miRNAs of *O*. *glaberrima* as ogl-miR01 to ogl-miR335. Among them, though we did not find any family members i.e same mature miRNA with multiple precursors, but found 17 pairs of miRNAs originated from the same precursor, which were designated as 3-p and 5-p depending upon their location in the precursor. This kind of observation is well- documented in literature^43^, including wild relative of rice^24^. Further, to find out their conservation among the different plants, we did BLASTN analysis that generated 5 categories of miRNAs such as i) common (i.e. conserved) miRNAs e.g. ogl-miR88; ii) monocot specific e.g. ogl-miR08; iii) Oryza-genus specific miRNAs e.g. ogl-miR15; iv) specific to only *O*. *glaberrima*, e.g. ogl-miR129 and v) miscellaneous miRNAs with no distinct patterns, were also found (Table S13). This result indicates that even novel miRNAs have different degree of conservation among the different plant species depending upon their functional requirements.

## Discussion

Rice is traditionally salinity sensitive plants. Several molecules, such as structural and functional genes, QTLs, miRNAs as well as other non-coding RNAs are involved for salinity stress response^36,44^. High throughput sequencing is proven to be an efficient technique to understand molecular mechanisms of salinity stress tolerance. Non-coding RNAs, such as miRNAs, play vital roles to combat abiotic stresses. Several stress-responsive miRNAs have been discovered from rice^45^, under different abiotic stresses including salinity stress^46^. However, except few, majority of them are discovered from tolerant genotypes of cultivated taxa. On the contrary, wild relatives are good source for useful genes. Though there are few reports of miRNA from genus *Oryza* (Table 2), yet there is no report of any kind of miRNAs from *O*. *glaberrima*.

**Table 2:** Summary of the miRNAs discovered in different species of *Oryza*.

Name of species	miRNA	Method used	Reference
Seven AA-genomes, *O*. *brachyantha* and *O*. *punctata*	28 (polycistronic)	Sequence homology search	Baldrich *et al*.^62^.
*O*. *rufipogon*	512	High-throughput sequencing	Wang *et al*.^63^.
*O*. *coarctata*	433	High-throughput sequencing	Mondal *et al*.^24^.
*O*. *rufipogon*	505	High-throughput sequencing	Zhang *et al*.^64^.
*O*. *nivara*	276	*De novo* and homology	Zhang *et al*.^65^.
*O*. *barthi*	276		
*O*. *glumaepatula*	263		
*O*. *meridionalis*	251		
*O*. *longistaminata*	603	High-throughput sequencing	Zong *et al*.^66^.
*O*. *glaberrima*	845	High-throughput sequencing	In this study.

**Figure 8:**
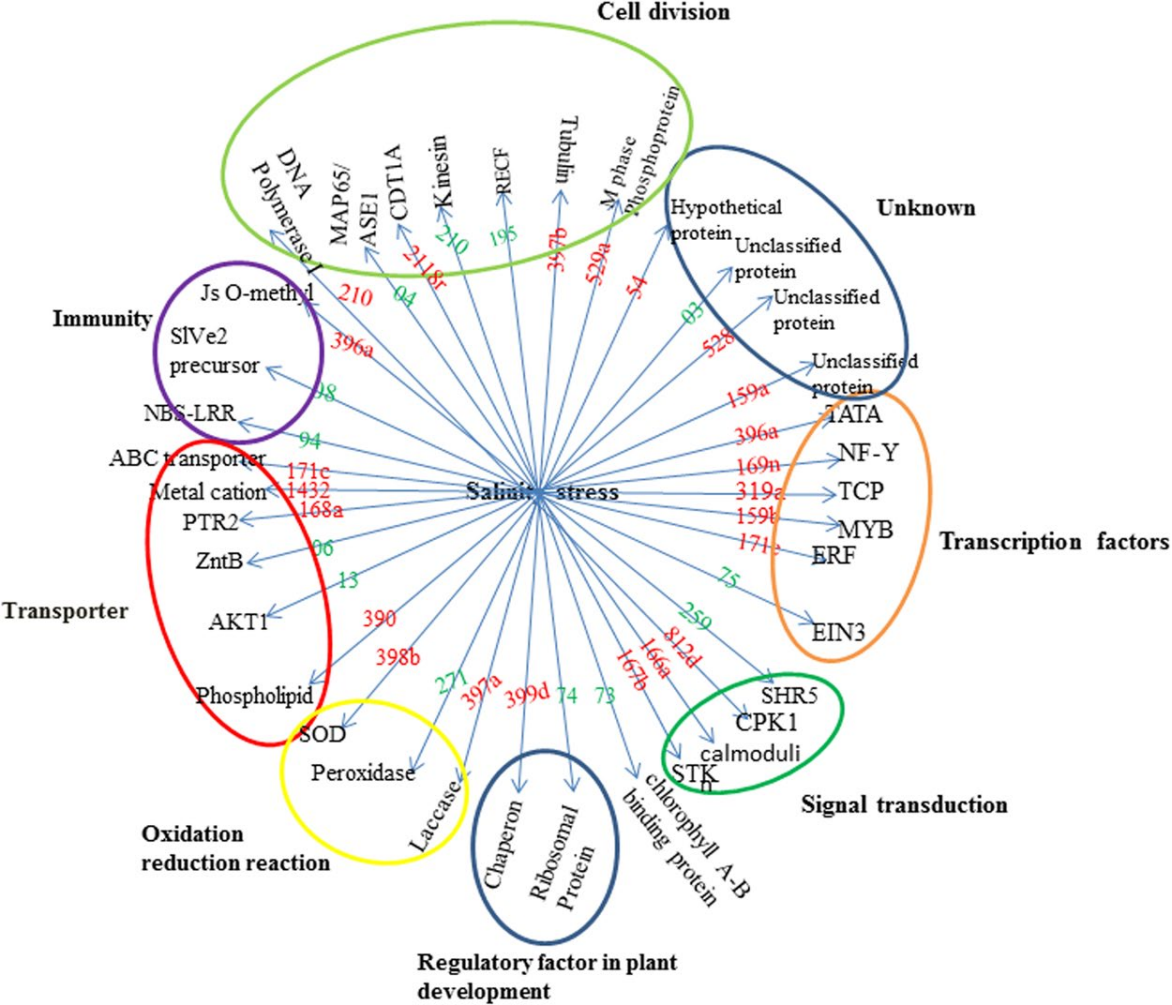
Pictorial representation of differentially expressed miRNAs along with respective targets under salinity stress. In internal circle with red color indicate known miRNAs and green colour indicate novel families. Outer circle represents the targets for respected miRNAs.

Thus, we discovered miRNAs from a salt tolerant genotype of *O*. *glaberrima*^47^, their precursors as well as targets. We also studied the relative expression of few modules in response to salinity stress. Finally, 29 conserved and 32 novel salt stress-responsive miRNAs were generated. Target genes were identified, found to be enriched in transcription factors, transporters, signal transducers proteins which are involved in oxidation-reduction reactions, photosynthesis and proteins with unknown functions (Fig. 8).

Transcription factors as targets may be crucial for plant tolerance to salinity stresses. Targets of several differentially expressed miRNAs were found to be NAC domain containing proteins (*NACs*), auxin response factor (*ARFs*), MYB domain proteins (*MYBs*) and nuclear factor Y subunit (*NF-Y*) (Tables S9 and S10) that are involved in the regulation of various stress-responsive genes^48^. Several targets are known to be involve as salinity stress response. For an example, *NF-Y* a target of miR169 modifies carbohydrate metabolism and cell elongation under salinity stress^39^. Similarly, miR159 which is found to target *MYB* and *ERF* TFs in the present study, widely modulates various abiotic stresses including salinity stress by integrating responses of environmental stimuli^49^.

Further, several ion-transporters or channels were targeted by miRNAs under salinity stress which indicates that these miRNAs are associated with ion homeostasis. The K^+^ transporter (AKT1), a predicted target in the present study modulates monovalent cation and pH homeostasis in plastids^50^. Thus, ogl-miR14-3p which target K^+^ antiporter is known to maintain Na^+^ and K^+^ homeostasis in *O*. *glaberrima*, an essential requirement of plant survival under salinity stress. Under salinity stress, overall growth and development at whole plant level is effected due to osmotic stress and later through cellular Na^+^ and Cl^−^ contents in cell that affects on K^+^ and Ca^2+^ uptake^51^, indicating that ion transporters play vital role in ion homeostasis under salinity tolerance. Cadmium and ABC transporter associated domain containing proteins which are found to be the targets in the present study, also known to be differentially expressed in rice under salinity indicating their involvement in salinity tolerance^51,52^. Similarly, an ABC transporter, a target of ogl-miR171c may play similar role in salinity adaptation of *O*. *glaberrima*.

Several genes related to cell division are also involved under salinity stress. It is well-known that salinity stress regulates cell division (Fig. 8). The primary response of salinity stress is the generation of ROS. Several enzymes that play important role as scavenger of ROS are found as targets due to salinity stress. Thus, we concluded that known miRNAs such as ogl-miR398b, ogl-miR397a and ogl-miR271 might play important role to provide salinity stress in this species as their targets are mostly enzymes involved in oxidation-reduction reactions.

Finally, it has been found that several uncharacterized proteins with unknown function are being targeted by novel as well as known miRNAs. These proteins could be specific to *O*. *glaberrima*. Such types of targets are well-reported in literature but it will be interesting to study in future to know their specific functions for salinity stress.

Salinity stresses also decrease the tolerance to biotic stresses in plants^53,54^. In the present study, ogl-miR94, ogl-miR01-5p and ogl-miR396a were differentially expressed under salinity stress which targeted disease resistance protein namely nucleotide-binding site (NBS)-leucine-rich repeat (LRR) protein, jasmonate O-methyltransferase and SlVe2 precursor respectively^55^ indicating that biotic stress related proteins were also induced under salinity stresses (Figs 8 and S10). Thus, abiotic and biotic stresses may share some common regulatory mechanisms and hence, salinity stress may make the plant susceptible to various diseases.

## Conclusions

We discovered 148 known and 335 novel miRNAs of *O*. *glaberrima* by NGS data. Further, through comparative genomics, we identified 246 conserved miRNAs from NCBI-SRA data. Relative expression, GO as well as KEGG pathway analysis revealed that several targets were involved in salinity stress response of *O*. *glaberrima*. miRNAs such as ogl-miR399i, ogl-miR396-5p, ogl-miR159b, ogl-miR398b, ogl-miR169a, ogl-miR10-3p, ogl-miR11, ogl-miR94 and ogl-miR172b were found to be salt-responsive. Among the targets, some are found to be transcription factors and protein-coding genes of some metabolic pathways that may be used in future to engineer the rice plants with better salinity tolerance. Overall, this study is an important document for salt responsive miRNA-target modules along with their putative molecular mechanisms of salt responsive changes in *O*. *glaberrima*.

## Materials and Methods

### Plant materials and salt treatment

RAM100 is a salt tolerant genotype of *O*. *glaberrima*^47^. Seeds of ‘RAM100’ were surface sterilized with 0.1% HgCl_2_ (w/v) and grown for 10 days in paper towel. After 10 days, seedlings were planted in pots and kept under green house conditions. The salt stress (EC of ~10 ds/m) with sodium chloride was imposed to 2-month-old seedlings for 48 h. Simultaneously, control samples (0.6 ds/m) of same age were treated with distilled water. After that, the leaf tissues from the stressed as well as control seedlings were immediately dipped in liquid nitrogen and kept at −80 °C for further use.

### Construction of small RNAs libraries

Summary of the work is presented in Fig. S1. Small RNA libraries were constructed following the protocol of Mondal *et al*.^24^. Small RNAs were purified from total RNA extracted from leaves of 2-month-old seedlings (Fig. 1). Equal amount of smallRNA from 3 replications were pooled for library preparation. Finally, two libraries per treatment were constructed, one from non-treated and another from salt-stressed leaves. The library quantified by Qubit Fluorometer, and quality was checked by High Sensitivity Bioanalyzer Chip (Agilent).

### Bioinformatics analysis

Initially, raw reads were removed for low-quality tags, tags with 3′ adaptor nulls, 5′ adaptor contaminants, or tags smaller than 18 nt, poly-A tags and insert nulls. Clean sequences then further filtered with non-coding RNAs such as rRNA, snRNA, repeat RNA, tRNA, and snoRNA. The remaining sequences (18 to 28 nt long) were taken to BLAST against the miRBase 21.0^25^ for identification of known miRNAs. Sequences with ≤2 mismatch bases were declared as known miRNAs, rest reads are defined as unique reads which were used to discover novel miRNAs as described by us previously^24^.

### Discovery of *O*. *glaberrima* miRNAs from NCBI database

All rice precursor sequences from miRbase 21.0 were used as input in SRA BLASTN against 6 smallRNA libraries [SRX055357 to SRX055362] of *O*. *glaberrima*. Identified sequences were used as input in Mfold^56^ for predicting the stem-loop structure which has been described above.

### Expression analysis of miRNAs

We analysed relative expression of novel and known miRNAs under salinity stress by using log_2_-ratios^57^. Initially, data was normalized to expression of transcript per million (TPM) [Normalized expression = (actual count of miRNAs/total count of clean reads)]. Additionally, fold-increase as well as *p*-values were also determined from the normalized expression. We described up regulated gene, if log_2_ Fold-Change ≥ 1 at *p ≤ *0.05 and down regulated gene, if log_2_ Fold-Change ≤ −1 at *p* ≤ 0.05. Fold change was calculated as: Fold - change = log_2_ (treatment/control) *p*-value.

### Gene ontology analyses of targets

For predicting the targets, psRNATarget with default parameters was used^58^, using *O*. *glaberrima* as the reference genome. Target cleavage validation was done using 5′- RACE-ready cDNAs with First Choice RLM-RACE Kit (Ambion, USA) as per our previous study^24^. Different primers are tabulated in Table S1. To investigate biological role of miRNAs, all the predicted targets of differentially expressed miRNAs (both known as well as novel) were used as input for KEGG enrichment analysis of KOBAS 2.0^59^ with a *p*-value of < 0.05. After that, putative targets as rice orthologs genes were taken for BLASTN search against rice as reference genome with AgriGO^60^ and were normalized with *p*-values (≤0.05).

### qPCR analysis of miRNAs and their targets

15 miRNAs as well as their targets were randomly selected to analyze the expression by qPCR. Around 1 μg smallRNAs were extracted from the leaf tissue of non-treated and salt-stressed (200 mM NaCl for 48 h) of *O*. *glaberrima* which were poly-adenylated and reverse transcribed with Mir-X miRNA First-Strand Synthesis kit (Clontech) as per the manufacturer’s instructions.

The diluted cDNA from previous step was PCR amplified with predicted mature miRNAs as forward primer and mRQ 3′ primer as reverse primer supplied with kit. 15 random primers (miRNAs) of 3 different categories were selected. They were, i) 5 each differentially expressed known miRNAs; ii) novel miRNAs and iii) miRNAs identified from public database. All the primers of qPCR experiment are listed in Table S1. For expression analysis of targets, gene-specific primers were designed and qPCR analysis was conducted in a 96-well plate using Roche 454 qPCR system (Roche, USA) as described previously^24^. For determination of relative expression, ‘comparative Ct method’^61^ was used. Normalization of Ct value was done using U6 snRNA as internal control primer that was supplied with the kit. For each reactions, 2 biological replicates each with 2 technical replicates were taken. For statistical analysis, SAS software of JMP Genomics (SAS Institute, NC, USA) was used. For targets, we used actin gene (ORGLA11G0038100) as reference gene.

### Data access

All the sequences were deposited in NCBI as Bioproject ID: PRJNA313396.

## Electronic supplementary material

Supplementary Figures 

Supplementary Data
