# Supplementary material for: Discovery of microRNA-target modules of African rice (Oryza glaberrima) under salinity stress
Source: Sci Rep. 2018 Jan 12;8:570. doi: 10.1038/s41598-017-18206-z (PMC5766505; doi:10.1038/s41598-017-18206-z)
Supplement: Supplementary file 1 — Supplementary Figures [file 41598_2017_18206_MOESM1_ESM.pdf]

Discovery of microRNA-target modules of African rice (*Oryza glaberrima*) under salinity stress.

Tapan Kumar Mondal*, Alok Kumar Panda[1], Hukam C Rawal[1], Tilak Raj Sharma[1]

Division of Genomic Resources, ICAR-National Bureau of Plant Genetic Resources, Pusa, IARI Campus, New Delhi- 110012, India; [1]ICAR-National Research Centre on Plant Biotechnology, L.B.S. Building, IARI Campus, New Delhi-110012.

[email*: mondaltk@yahoo.com] Tel: 91-011-25848783; Fax: 91-011-25843984

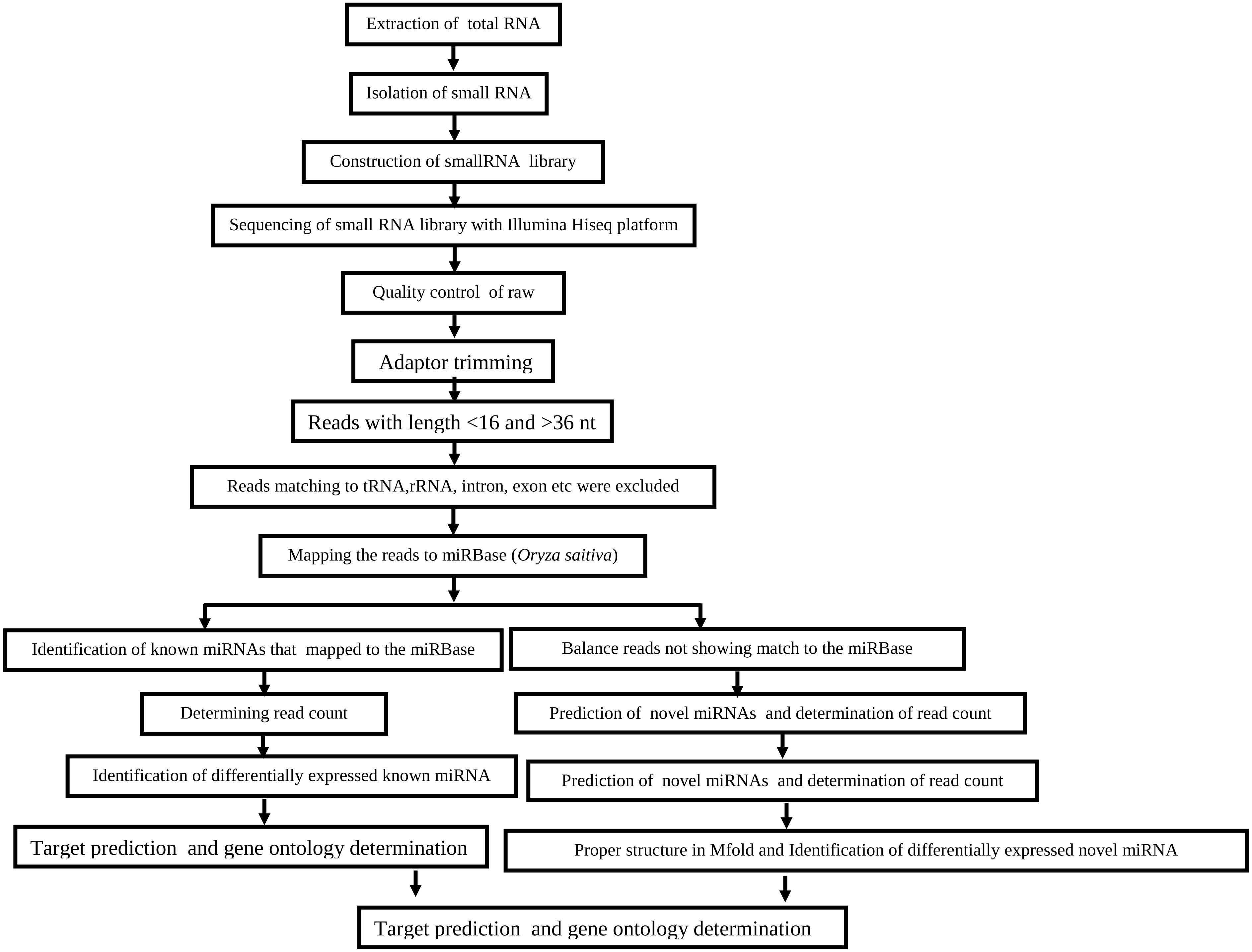

Fig S1. Outline of the Work of small RNA discovery and analysis of *O. glaberrima*

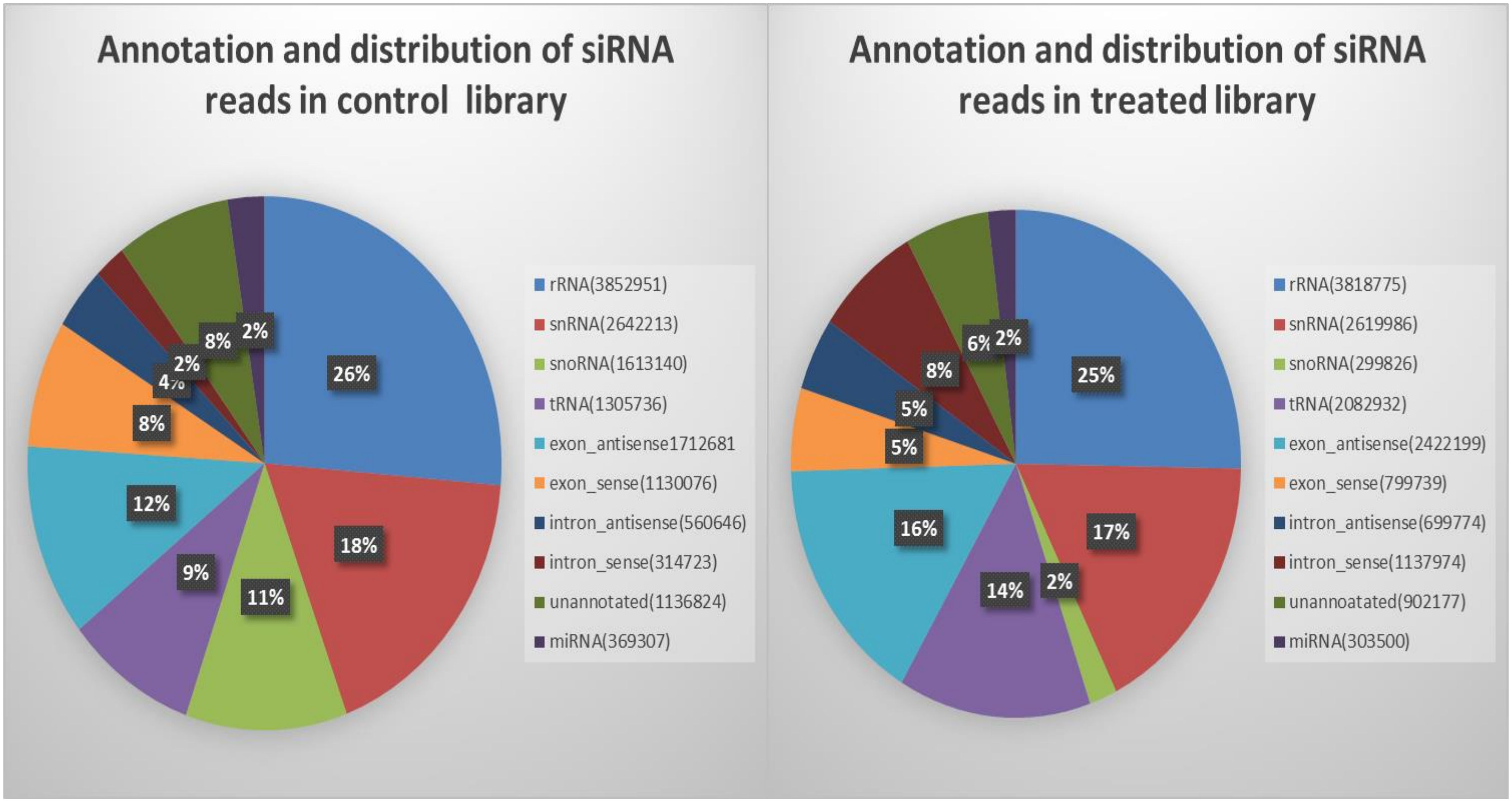

Fig S2. Annotation and distribution of mRNAs in control and salt-treated library.

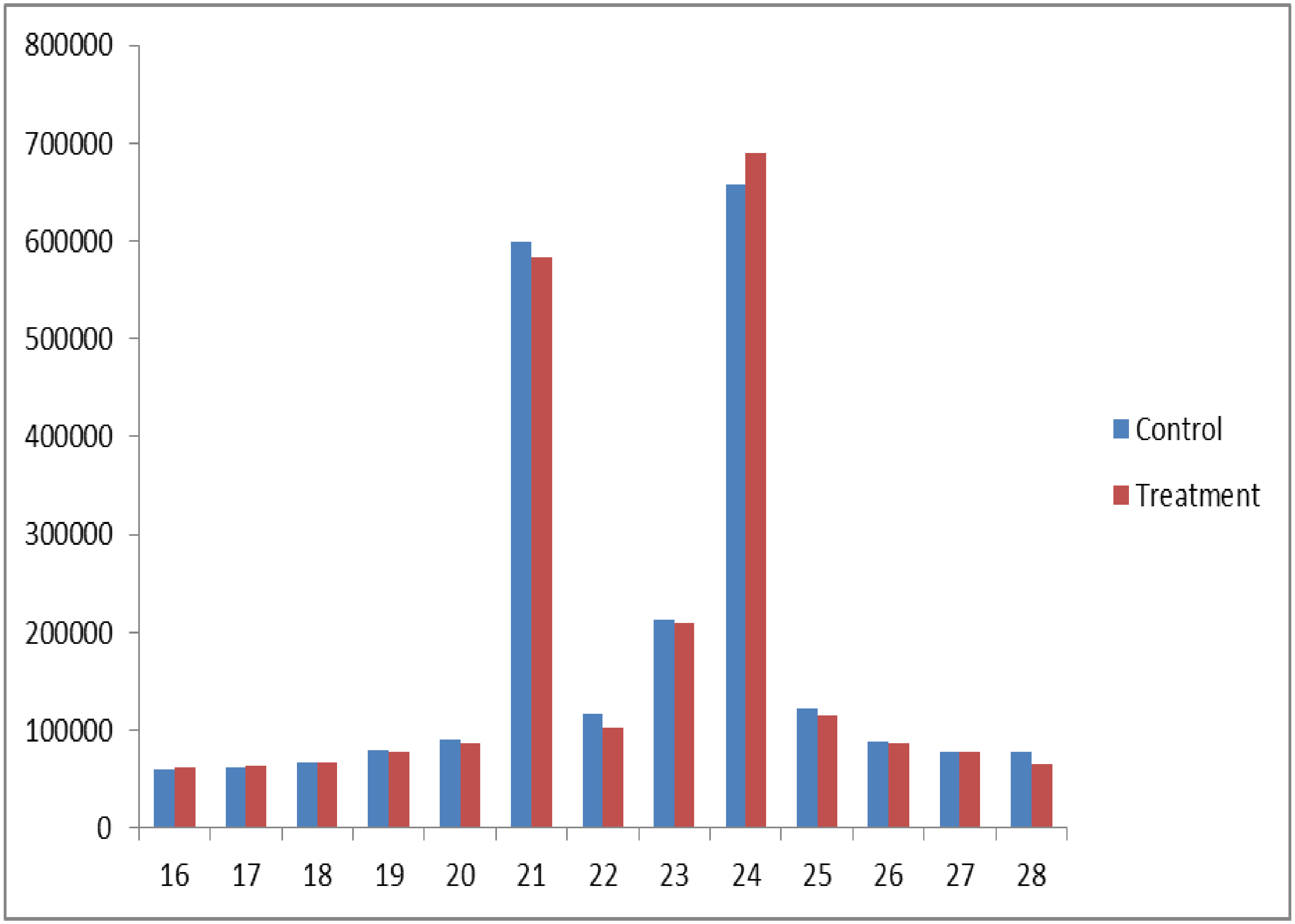

Fig S3. Size distribution of small RNA clean reads.

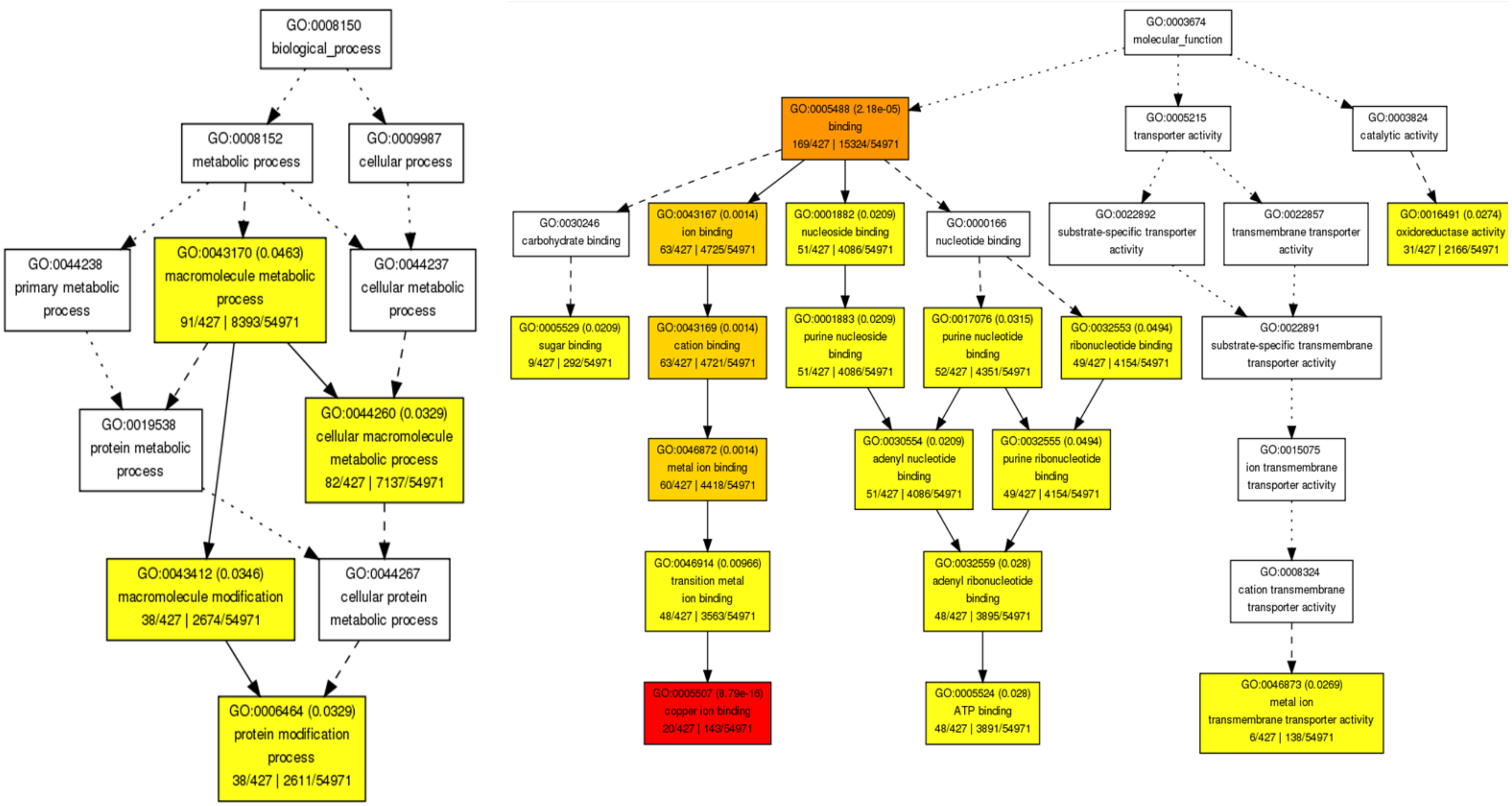

Fig S4. Gene Ontology (GO) analysis of salt-stressed target genes of *O. glaberrima* with AgriGO

Table S1: Primers used in the present study

miRNA	Nature	Regulation as illumine sequencing	miRNA specific forward primer	Target ID	Target specific forward primer (5'-3')	Target specific reverse primer (5'-3')
ogl-miR399i	Known	Up	TGCCAAAGGAGAGCTGCCCTG	LOC_Os04g55230	CTTGATGAAGCTGGAGAACATGCCTG	GACAGAGCAGTTACTGTCAGAGTCTTCCTTC
ogl-miR396c-5p	Known	Up	GGTCAAGAAAGCTGTGGGAAG	LOC_Os03g51970	GTTTCCCTTCAACACTTTGTGGACTGG	CATCCAGTTTGCTTCTCCCTGGTTAAC
ogl-miR159b	Known	Up	TTTGGATTGAAGGGAGCTCTG	LOC_Os01g12700	TCTGCACCCCAGTTTTCATATGCTTC	CTCCATCTGCAAAGCATCAGTTGTGG
ogl-miR398b	Known	Down	TGTGTTCTCAGGTCGCCCCTG	LOC_Os07g46990	CCACACTTCAATCCTACTGGGAAGGAAC	CCAGCATTTCCAGTGGTCTTGCTAAG
ogl-miR169a	Known	Down	CAGCCAAGGATGACTTGCCGA	LOC_Os12g42400	GACAGGGGTGTCCCTGAAATTCTGAAC	CATTCACATAAATTGGTGCATCAGCTG
ogl-miR10	Novel	Up	ATCAATAGTAGATGGAGGGAGT	LOC_Os07g36780	GACCATCCAACATGTCAATCTGGTTCG	CCAAAAGTATGTTATCTGGCTTAACATCACAG
ogl-miR11	Novel	Up	ATAAACCATAAGCTCCACTCAA	LOC_Os03g62820	GCAGATCTGAAAAATGCATCAAGCTTAAC	GCTCTTCGATCATCACCCTCCGTTG
ogl-miR180	Novel	Down	CAGCTGCCGGTTCATGGATACCT	LOC_Os02g11960	CAAGGGTGATGGACAATATGTTCCCTG	GTTCATCGTTGATGAGCCACAAACTGTC
ogl-miR175	Novel	Down	TTGCTCGAACGCCGGGTCGCCG	LOC_Os04g25410	CATATGGTGCTCTTCTTAACTGTTACTGCAAAG	ACACGACCATCCCGTTTCATCTCTTC
ogl-miR94	Novel	Down	TTTGCAAAGTTTGAGATTTGAC	LOC_Os04g41950	GGGCCTTAGTATCCTGTGTCACCCAAC	TTAGCTTTAGCTTTTGCAGTGCTTAAGCC
Ogl-169o	*In silico*	Up	TAGCCAAGAATGACTTGCCTA	LOC_Os03g48970	ACTGATCAAGAAAAGCATCATGCAACATC	TGCTTAGCATTTACATAAACAGGCTCCTCAG
Ogl-319b	*In silico*	Up	UUGGACUGAAGGGUGCUCCC	LOC_Os12g42190	CTCGGCGTCGACCAGCCGAGCAAG	TGCTGCTGTTGCTGCAATGGCTTGC
Ogl-172a	*In silico*	Up	AGAAUCUUGAUGAUGCUGCAU	LOC_Os05g03040	TACGAGGAGGACATGAGGCAGATGAAG	CCATTGCATTTGATCGCAGCCTTATC
Ogl-399b	*In silico*	Down	UGCCAAAGGAGAAUUGCCCUG	LOC_Os1g41650	GTATTCAAAGTGTCTGTCGGCTGAAGATG	CACTCTTCACAATGTGTGCATGAATGC
Ogl-169e	*In silico*	Down	UAGCCAAGGAUGACUUGCCGG	LOC_Os03g29760	ACACACCTCACCACAATCAGTGTTTGTC	AATGGCATCCTAGAGTTTGCAGCACC
Outer primer for 5'RACE-PCR			CTGAAGACTCTTAAGCTCAACAATG	TIR1 orthologue from *O. glaberrima*		
Nested primer for 5' RACE-PCR			ACTTAATCTGAGTTATGCTACTGTGC	TIR1 orthologue from *O. glaberrima*		
NA= Not available						
